# Therapeutic effect of Intra-Tympanic Dexamethasone–Hyaluronic Acid Combination in Sudden Sensorineural Hearing Loss

**Published:** 2017-09

**Authors:** Mehrdad Rogha, Amin Kalkoo

**Affiliations:** 1 *Department of Otorhinolaryngology, Isfahan University of Medical Science, Isfahan. Iran.*

**Keywords:** Dexamethasone, Hyaluronic acid, Intra tympanic injection, Sudden sensorineural hearing loss

## Abstract

**Introduction:**

Hearing loss is fairly a common disorder which is usually treated with corticosteroids via systemic administration and/or intra-tympanic injection. This study aimed to compare the effectiveness of intra-tympanic injections of dexamethasone with its combination with hyaluronic acid in patients with sudden sensorineural hearing loss.

**Materials and Methods:**

In this clinical trial, 40 patients were randomly assigned to two groups; in the first group, 20 patients received 2.4 mg intra-tympanic dexamethasone, while in the second group patients received injections of 2.4 mg of dexamethasone plus 2 mg of hyaluronic acid in combination. Patients in both groups were injected every other day to a total of three injections. The hearing status of patients was evaluated by pure tone audiometry (bone conduction threshold) before and 2 weeks after the intervention.

**Results:**

Assessment of hearing threshold before and after treatment in the two groups showed a significant difference between hearing thresholds at frequencies of 4,000 to 8,000 Hz (P<0.001). The difference at other frequencies was not meaningful; however, in general, we found a better therapeutic effect in patients who received the combination of dexamethasone and hyaluronic acid.

**Conclusion:**

A combination of dexamethasone and hyaluronic acid in patients with sudden sensorineural hearing loss may be more effective than dexamethasone alone. Because hyaluronic acid lacks certain side effects, and also makes it possible to reduce the steroid dose, we recommend the use of this combination in the treatment of patients with sudden sensorineural hearing loss.

## Introduction

In sudden sensorineural hearing loss, a person with normal or near-normal hearing loses his or her hearing in a short period of time, and maximally within 3 days. The defect can be partial or total. By definition, the drop in hearing should be at least 30 dB in three consecutive frequencies. The incidence of the disorder is estimated at one in 5,000 population per year (1-3). If left untreated, 31–65% of cases may recover spontaneously, within 2 weeks of the appearance of the symptoms (4,5). There are some therapeutic modalities for the treatment of this disorder, such as oral, intravenous and intra-tympanic corticosteroids, vasodilators, antiviral drugs and hyperbaric oxygen (6-11). While some studies have noted that systemic high-dose corticosteroid therapy is the most beneficial method of treatment (12-14), another study showed that intravenous treatment by corticosteroids and pentoxifylline resulted in complete remission of signs and symptoms (15). Due to the well-known side effects of systemic corticosteroids and the lack of appropriate response to existing treatments, intra-tympanic injection is increasingly being used as a treatment modality, leading to a higher concentration of corticosteroids in the perilymph fluid (16-18). In this type of therapy, the steroid crosses over the round window valve and enters into the cochlea to induce its therapeutic effect. Since the cochlea is affected locally, the patient does not suffer the harmful side effects of systemic steroids, and therefore this type of therapy is more accepted nowadays. Some studies have also shown the benefit of this type of treatment in those who have not responded to initial treatment with systemic steroids (19-22).

The expected time for recovery is usually about 2 weeks to allow response to treatment, while some patients treated with systemic steroids for 2 weeks have shown no improvement in hearing thresholds. The mechanism of action for steroid therapy in this disease is not yet fully known, but previous studies have shown that corticosteroid effects on the cochlea include the reduction of ischemia, increase in blood flow and reduction of inflammation (23,24). It is postulated that the increase in blood flow occurs 30 seconds after injection, while there is still no apparent effect on patient hearing. A 2011 study revealed that intra-tympanic steroids improve hearing loss significantly at the low-frequency range (0.5–1 kHz) (21). Treatment with a combination of hyaluronic acid and dexamethasone has also been used for the treatment of Meniere's disease with hearing loss at low frequencies (25). In animal studies, it has been shown that hyaluronic acid can increase the permeability of the round window valve and thereby increase the concentration of the steroid in the inner ear (26-28). In addition, the mixture of these two substances in patients with hearing loss at high frequencies (above 3 kHz) and low frequency (0.5–1 kHz) may be useful and improve symptoms (29,30). Hyaluronic acid not only increases the permeability of the round window, but affects steroid distribution in the inner ear, and its effect on high-frequency hearing loss may be due to its effect on the hair cells (29). Therefore, this study aimed to determine the effectiveness of dexamethasone with hyaluronic acid inside the ear in patients with sudden sensorineural hearing loss.

## Materials and Methods

This double-blind clinical study was conducted at Ayatollah Kashani Hospital in Isfahan, Iran in 2015 in patients with a diagnosis of sudden sensorineural hearing loss. Inclusion criteria consisted of unilateral hearing loss (occurring within 72 hours), a hearing threshold average greater than 50 dB, appearance of symptoms within 7 days from the intervention, age between 18 and 80 years, and provision of consent to participate in the study. Exclusion criteria were history of acoustic tumor, middle ear diseases, autoimmune diseases, vestibular problems (including Menier disease), cardiovascular disorders, diabetes mellitus and previous ear surgery. A patient’s refusal to attend follow-up visits on time as well as any limitation on injecting into the intra-tympanum via the external auditory canal, such as stenosis, anatomical deformities or external and middle ear infections, were considered exclusion criteria. Bone and air conduction pure tone audiometry were performed in all patients before treatment. Taking into account the 95% confidence level, 80% statistical power, improvement in hearing equivalent to 0.5 and a desired 0.5 difference between the two groups, it was calculated that 16 patients were required in each study group. For greater reliability, 20 patients were assigned randomly into each group. All patients initially underwent an ear examination and audiometric assessment. The first group received dexamethasone and the second group was injected with dexamethasone plus hyaluronic acid (Hyaluron Hexal 20 mg/20 ml) in combination. Injectable solutions were previously prepared and coded. For Group 1, 0.6 ml of dexamethasone solution (8 mg in 2 ml), equivalent to 2.4 mg of dexamethasone plus 0.2 ml distilled water, was loaded into an insulin syringe. For Group 2, 0.6 ml of dexamethasone solution (8 mg in 2 ml), equivalent to 2.4 mg of dexamethasone, plus 0.2 ml hyaluronic acid 0/1%, equivalent to 2 mg, were similarly loaded. For local anesthesia, the superior part of the anterior and posterior-external auditory canal walls were injected with xylocaine 2%.

Positioning the patient in the supine position with the head rotated upward and at a 45° angle toward the opposite ear allowed the round window niche to be set at the deepest and the Eustachian tube at the highest level of the tympanum cavity. Then, under microscopic vision, the prepared solution in the insulin syringe was injected into the posterior inferior tympanic membrane via a 26-gage needle. This procedure was repeated three times, with injections every other day. After 2 weeks, a second audiogram was obtained and compared with the first. A 30-db improvement in bone conduction threshold in pure tone audiometry was considered a significant improvement, while improvements of 10–30 dB and <10 dB were considered as partial recovery and non-recovery, respectively. Finally, data were entered into SPSS version 23 and analyzed using the Chi-square test, T-test, paired T-test and analysis of variance (ANOVA) with repeated observations.

## Results

In this study, 40 patients with sudden deafness were divided in two groups of 20 and received dexamethasone alone or dexamethasone plus hyaluronic acid, respectively. Table 1 shows demographic and clinical variables between the two groups. Based on this table, the distribution of age, sex, symptoms, duration of illness and the affected ear in the two groups was not significant. 

**Table 1:** Distribution of demographic and clinical characteristics in two groups

**Variables**		**Groups**		**P**
		**Dexamethasone **	**Dexamethasone with hyaluronic acid**	
Mean age (years)		42.8±9.4	42±14.2	0.83
Sex N (%)	Male	13 (65)	16 (60)	0.29
	Female	7 (35)	4 (20)	
Comorbidity N (%)	No	2 (10)	1 (5)	0.31
	Headache	0 (0)	2 (10)	
	Nausea	18 (90)	17 (85)	
Site	Left	9 (45)	7 (35)	0.52
	right	11 (55)	13 (65)	
Duration of disease (day)		4.15±1.3	4.7±1.1	0.16
			

Table 2 shows the mean and standard deviation values of hearing bone conduction threshold at different frequencies before and after treatment in both groups. Based on the paired T-test, the mean hearing threshold at all frequencies after treatment in both groups was significantly improved. 

**Table 2:** Mean and standard deviation of bone conduction thresholds before and after treatment in both groups

**Frequency**	**Time**	**Groups**		**P** *
		**Dexamethasone **	**Dexamethasone with hyaluronic acid**	
250	Before	60.5±22.8	51.5±23.2	0.22
	After	39±18.3	43±13	0.43
	P**	0.028	0.004	0.62
500	Before	46±17.3	49±11.3	0.32
	After	46±17.3	49±11.3	0.52
	P**	0.005	0.13	0.67
1000	Before	67±26.4	66±16.4	0.89
	After	53±17.8	52±12.4	0.84
	P**	0.002	<0.001	0.86
2000	Before	70±15.8	75.5±22.1	0.37
	After	53.8±12.2	56.5±19.5	0.6
	P**	<0.001	0.003	0.37
4000	Before	71±16.7	80.5±17.3	0.09
	After	54.5±14.5	68.5±13.1	0.003
	P**	<0.001	0.001	0.012
80000	Before	69.5±19.6	81.5±16.9	0.06
	After	51.5±19.8	69.5±13.6	0.002
	P**	<0.001	<0.001	0.006

* Difference between groups in any point time based on T-test

** Difference within each group based on paired T-test

*** mean changes between the two groups based on repeated measures ANOVA

**Fig1:**
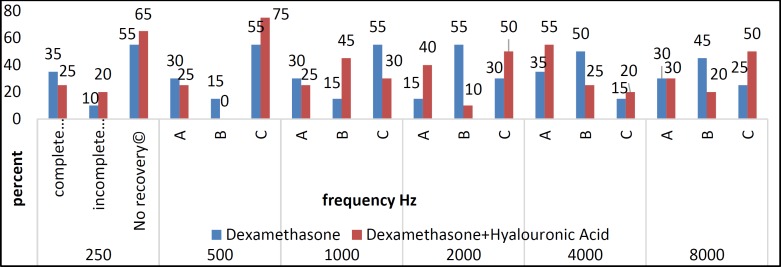
Percentage of the patients with hearing improvement after treatment in two groups

A comparison of hearing thresholds before and after treatment between the two groups using the T-test showed that the average hearing thresholds at frequencies of 4,000 and 8,000 Hz was statistically meaningful. Finally, the ANOVA with repeated observations showed that the hearing threshold difference between the two groups was significant and that improvement of hearing was greater in the hyaluronic acid plus dexamethasone group than in the dexamethasone-only treatment group. Figure 1 shows the frequency of remission status after treatment in the two groups receiving dexamethasone alone and dexamethasone plus hyaluronic acid. According to the graph, the percentage improvement at frequencies of 4,000 and 8,000 kHz was higher in the group receiving dexamethasone plus hyaluronic acid. According to the Mann-Whitney test, the recovery rates at frequencies of 2,000 and 4, 000 kHz were significantly different, but no significant difference between the two groups was seen at other frequencies.

## Discussion

At the present time, the treatment of sudden hearing loss is considered a major challenge in otolaryngologic practice. Further, because of the relatively high prevalence of the disease and its impact on patient quality of life, there is a need for a therapeutic modality with few complications and a general acceptance by practitioners and researchers. In a 2011 study by Rauch et al, in which 250 subjects were followed for 6 months, both oral and intra-tympanic corticosteroid were shown to have a meaningful therapeutic effect in sudden hearing loss. In another study, conducted in Italy by Dalen et al, use of intra-tympanic corticosteroid as a salvage therapy after failure of oral corticosteroids was shown to be an effective modality in the treatment of sudden hearing loss.

We believe that the addition of the highly viscous hyaluronic acid may prolong the time the corticosteroid remains within the middle ear cavity, which, in turn, may lead to a better therapeutic effect. It is noteworthy that there are few studies on the effect of hyaluronic acid in combination with a corticosteroid in this type of treatment for sudden hearing loss. Many studies have shown that treatment with corticosteroids is currently the best supporting treatment for sudden deafness. In addition to the greater effectiveness of the drug when used locally, this method also prevents the systemic side effects of oral or parenteral prescription. Other studies have also shown that hyaluronic acid can be helpful in the treatment of sudden deafness (29). Therefore, we decided to compare the effect of topical application of dexamethasone and hyaluronic acid as a mixture for the treatment of sudden deafness against single drug therapy with dexamethasone.

In this study, there were no significant differences between the two groups in terms of demographic and clinical variables at baseline, eliminating any confounding effect of these factors on hearing and recovery of the patients. The results showed that adding hyaluronic acid to dexamethasone in patients had a significant effect on hearing and increased the speed of recovery, especially at high frequencies. Although the process had non-significant changes in hearing threshold at low frequencies, the improvement at these frequencies was numerically better in the group receiving dexamethasone plus hyaluronic acid. In a study conducted in 2011 by Ulfendahl, it was shown that introduction of corticosteroid within the tympanic cavity can significantly improve symptoms in low frequency sounds (500 Hz) (21). The mixture of hyaluronic acid and dexamethasone can also be used for the treatment of Meniere's disease with a low frequency hearing loss, an effect that was confirmed in study by Selivanona (25). Animal studies have also shown that hyaluronic acid increases the permeability of the round window membrane and thereby increases the concentration of the steroid drug in the inner ear (26,27). The mixture of these two substances in patients with hearing loss at high frequencies (1,500–3,000 Hz) and low frequency (500 Hz) can be beneficial and improve symptoms (29).

## Conclusion

Considering that hyaluronic acid has no major side effects, is able to reduce essential steroid dosage, and results in better treatment outcomes, we recommend the combination of dexamethasone and hyaluronic acid as an effective therapeutic modality in patients with sudden sensorineural hearing loss.
